# Tumor stiffness measured by 3D magnetic resonance elastography can help predict the aggressiveness of endometrial carcinoma: preliminary findings

**DOI:** 10.1186/s40644-021-00420-8

**Published:** 2021-08-28

**Authors:** Linqi Zhang, Xi Long, Mayidili Nijiati, Tianhui Zhang, Mengsi Li, Ying Deng, Sichi Kuang, Yuanqiang Xiao, Jie Zhu, Bingjun He, Jingbiao Chen, Phillip Rossman, Kevin J Glaser, Sudhakar K Venkatesh, Richard L Ehman, Jin Wang

**Affiliations:** 1 Department of Radiology, the Third Affiliated Hospital of Sun Yat-sen University, Guangzhou, People’s Republic of China; 2 Department of Radiology, Meizhou People’s Hospital (Huangtang Hospital), Meizhou Hospital of Sun Yat-sen University, Meizhou, People’s Republic of China; 3 Department of Radiology, The First People’s Hospital of Kashi Area, Kashi, People’s Republic of China; 4 Department of Radiology, Mayo Clinic, Rochester, MN United States

**Keywords:** Magnetic resonance elastography (MRE), Tumor stiffness, Endometrial carcinoma (EC), Tumor aggressiveness

## Abstract

**Background:**

Preoperative evaluation of aggressiveness, including tumor histological subtype, grade of differentiation, Federation International of Gynecology and Obstetrics (FIGO) stage, and depth of myometrial invasion, is significant for treatment planning and prognosis in endometrial carcinoma (EC). The purpose of this study was to evaluate whether three-dimensional (3D) magnetic resonance elastography (MRE) can help predict the aggressiveness of EC.

**Methods:**

From August 2015 to January 2019, 82 consecutive patients with suspected uterine tumors underwent pelvic MRI and MRE scans, and 15 patients with confirmed EC after surgical resection were enrolled. According to pathological results (tumor grade, histological subtype, FIGO stage, and myometrial invasiveness), the patients were divided into two subgroups. The independent-samples t-test or Mann-Whitney U test was used to compare the stiffness between different groups. The diagnostic performance was determined with receiver operating characteristic (ROC) curve analysis.

**Results:**

The stiffness of EC with ≥ 50 % (*n* = 6) myometrial invasion was significantly higher than that with < 50 % (*n* = 9) myometrial invasion (3.68 ± 0.59 kPa vs. 2.61 ± 0.72 kPa, *p* = 0.009). Using a stiffness of 3.04 kPa as a cutoff value resulted in 100 % sensitivity and 77.8 % specificity for differentiating ≥ 50 % myometrial invasion from < 50 % myometrial invasion of EC. The stiffness of poorly differentiated EC (n = 8) was significantly higher than that of well/moderately differentiated EC (*n* = 7) (3.47 ± 0.64 kPa vs. 2.55 ± 0.82 kPa, *p* = 0.028). Using a stiffness of 3.04 kPa as a cutoff value resulted in 75 % sensitivity and 71.4 % specificity for differentiating poorly differentiated from well/moderately differentiated EC. The stiffness of FIGO stage II/III EC was significantly higher than that of FIGO stage I EC (3.69 ± 0.65 kPa vs. 2.72 ± 0.76 kPa, *p =* 0.030). Using a stiffness of 3.04 kPa as a cutoff value resulted in 100 % sensitivity and 70 % specificity for differentiating FIGO stage I EC from FIGO stage II/III EC. The tumor stiffness value in type II (n = 3) EC was higher than that in type I (n = 12) EC (3.67 ± 0.59 kPa vs. 2.88 ± 0.85 kPa), but the difference was not significant (*p* = 0.136).

**Conclusions:**

Tumor stiffness measured by 3D MRE may be potentially useful for predicting tumor grade, FIGO stage and myometrial invasion of EC and can aid in the preoperative risk stratification of EC.

## Background

Endometrial carcinoma (EC) is one of the most common primary malignant tumors in women, and its incidence is steadily rising worldwide [1]. Preoperative evaluation of aggressiveness, including tumor histological subtype, grade of differentiation, Federation International of Gynecology and Obstetrics (FIGO) stage, and depth of myometrial invasion, is essential in planning the surgical procedure and determining whether sampling of pelvic and paraaortic lymph nodes should be performed [2, 3]. Previous studies have shown that the presence of high tumor aggressiveness in hysterectomy specimens at surgicopathological staging is associated with an increased risk of lymph node metastases, tumor recurrence, and distant relapse in EC patients [4]. Thus, these patients may benefit from primary radical hysterectomy with removal of the uterus, parametrium and lymphadenectomy [3, 4]. Endometrial biopsy or dilatation and curettage are used to evaluate the aggressiveness of EC. However, these methods require invasive tissue sampling, specialized equipment and analysis, and they are limited by sampling bias and possible complications [5]. Accordingly, the development of a noninvasive and accurate imaging modality to evaluate the tumor aggressiveness of EC would be useful for deciding the therapeutic strategy and predicting prognosis. However, there is no consensus regarding the optimal imaging technique for the prediction of tumor aggressiveness. Due to its high tissue contrast resolution and reproducibility, magnetic resonance imaging (MRI) is the preferred imaging modality for the preoperative evaluation of EC [6, 7]. Conventional pelvic MRI is highly sensitive for the detection of the primary tumor but typically has low specificity for determining tumor histological subtype, grade of differentiation, and depth of myometrial invasion [7, 8]. In addition, the interobserver variability between radiologists in the interpretation of the diagnostic images also represents a source of inaccuracy [9].

Recently, magnetic resonance elastography (MRE) has emerged as a noninvasive technique for evaluating tissue viscoelastic properties by measuring the shear waves produced by a vibrating mechanical transducer. As an alternative to biopsy and to enable noninvasive visualization and quantification of stiffness, MRE has been used successfully to estimate the stage of hepatic fibrosis [10]. Recently, MRE has been introduced as a method to evaluate whole-tumor stiffness for various cancers, such as hepatocellular carcinoma, brain tumor, and breast and prostate cancer [11–18]. Previous studies have indicated that MRE may be potentially useful for predicting the tumor grade and prognosis of hepatocellular carcinoma [11, 18]. Regarding MRE applied in the uterus, one previous study investigated the viscoelasticity of the uterus measured by MRE in healthy volunteers considering individual variations over the menstrual cycle [19]. Another study revealed that MRE is a feasible technique for studying the in vivo mechanical properties of uterine leiomyomas [20]. However, to our knowledge, there are no data on MRE assessment of malignant uterine tissue.

Therefore, the purpose of this study was to evaluate the feasibility of transpelvic 3D MRE of the uterus and to assess the diagnostic performance of 3D MRE in predicting tumor aggressiveness, including histological subtype, tumor grade, FIGO stage, and myometrial invasion of EC.

### Methods

## Materials and methods

**Fig. 1:**
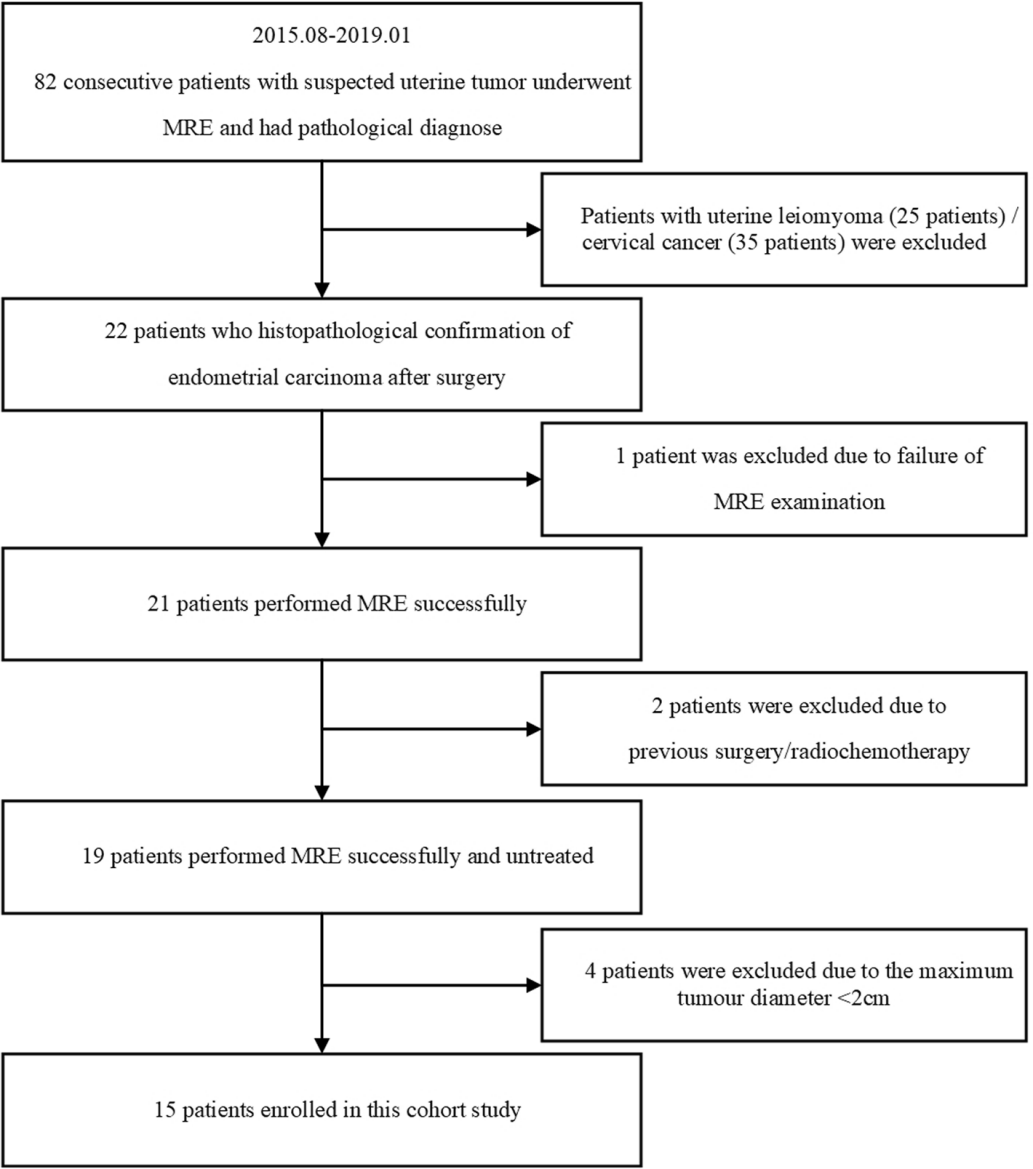
Flow chart of the patient selection process

This retrospective study was approved by our institutional review board, and all patients provided informed consent. From August 2015 to January 2019, 82 consecutive patients with suspected uterine tumors underwent pelvic MRI and MRE scans. Among them, 22 patients who had histopathological confirmation of EC after surgery were selected. Seven patients were excluded because (1) the MRE examination failed (n = 1) due to obesity (thickness of subcutaneous fat was 3.75 cm), and the images could not be used for quantitative analysis; (2) they had a history of previous radiotherapy/chemotherapy (n = 2); and (3) their lesion had a maximum diameter of < 2 cm (n = 4). Finally, 15 patients with EC were enrolled in this cohort study. Figure 1 details the patient selection process with a flow chart. All patients underwent hysterectomy within 1 month after MR examination.

### MRI and MRE protocol

MRI was performed on a 3.0T scanner (GE, Discovery MR750, Waukesha, WI) with 8-channel phased-array torso coils (GE, Waukesha, WI) for signal acquisition. MRE was performed with a pneumatic driver developed by Mayo Clinic (Rochester, MN, USA). The driver was placed above the pelvis. Continuous acoustic vibrations at 60 Hz, which were transmitted from an active driver to the passive driver via a flexible vinyl tube, were used separately to produce propagating shear waves in the cervix. A test vibration was first applied to allow the patient to become familiar with the vibration. A free-breathing, multislice, EPI, 3D-MRE sequence was used. The acquisition parameters included repetition time = 1334 msec, echo time = 52 msec, slice thickness = 3 mm, interslice gap = 0 mm, number of slices = 20, FOV = 240 mm, acquisition matrix = 80 × 80, superior-inferior spatial saturation bands, and parallel imaging acceleration factor = 2. The total imaging time for MRE was approximately 80 s. Diffusion-weighted imaging (DWI) was performed using a respiratory-triggered single-shot spin echo echoplanar imaging sequence in the transverse plane before contrast-enhanced imaging, with 2 *b* values (*b* = 0 and 800 s/mm^2^).

### MR elastogram analysis

The MRE-acquired displacement fields were processed using a 3D direct inversion (DI) of the Helmholtz wave equation after applying the curl operator to calculate a stiffness map over the uterine volume. The reconstruction matrix was 256 × 256 × 20. The processing steps were applied automatically to generate quantitative images of tissue shear stiffness maps in units of kilopascals (kPa).

In each case, the mean stiffness of the EC was calculated using a manually specified region of interest (ROI). The ROIs were drawn by two radiologists (X.L., T.Z.), both of whom were highly experienced in pelvic MRI with more than 5 years of experience. The ROIs excluded tumor edges, areas of significant wave interference and any other artifacts seen in the magnitude and wave images. The tumor ROI was drawn on the magnitude images with reference to conventional MR images. Then, the ROIs were copied to the stiffness map, which gave the stiffness values in kPa. The mean stiffness in kPa within the ROIs was reported. Apparent diffusion coefficient (ADC) maps were automatically generated from each DWI image by MRI system software (version AW 4.6, GE Healthcare).

### Surgery and pathological analyses

Patients were staged on the basis of the histopathological findings following surgical resection, according to the revised FIGO staging system (2009) [21]. All original uterine specimens were reviewed by a gynecological oncological pathologist with more than 20 years of experience in uterine pathology who was blinded to the imaging findings. Surgical specimens were sectioned along the longitudinal plane of the uterus. Deep myometrial invasion was defined as a tumor invading ≥ 50 % of the myometrium. The depth of myometrial invasion, tumor histological subtype and grade of differentiation were estimated grossly and confirmed microscopically according to standard procedures.

### Statistical analyses

Interobserver reproducibility for the ROI measurements of the two readers was assessed by using the interclass correlation coefficient (ICC: <0.40 poor, 0.41–0.60 moderate, 0.61–0.80 good, and 0.81–1.00 excellent correlation). The independent-samples t-test was used to compare the stiffness value between different groups with a normal distribution. The Mann-Whitney *U* test was used to compare the stiffness value between different groups with abnormal distribution. To characterize the diagnostic accuracy of tumor stiffness in the assessment of tumor aggressiveness, receiver operating characteristic (ROC) curves were implemented. By maximizing the Youden index, we determined the optimal cutoff values and corresponding sensitivities and specificities for distinguishing between different groups. Statistical analysis was performed with SPSS 22.0 (Chicago, IL). *p*-values < 0.05 were considered statistically significant.

## Results

### Patients

The mean patient age in the study sample (*n* = 15) was 50.20 ± 9.03 years (range 35–65 years). Among these patients, 6 (6/15, 40 %) were premenopausal and 9 (9/15, 60 %) were postmenopausal. Patient characteristics are detailed in Table 1. Applying the FIGO 2 criteria, 53.33 % (8/15) were stage IA with tumors invading < 50 % of the myometrium, 13.33 % (2/15) were stage 1B with ≥ 50 % myometrial invasion, 13.33 % (2/15) were stage II with cervical stromal invasion (including 1 patient with tumors invading < 50 % of the myometrium) and 20 % (3/15) were stage III with local or regional tumor spread. The histological subtype was endometrioid (type I EC) in 80 % (12/15) and type II EC in 20 % (clear cell in 1 patient, serous papillary in 1, and low-undifferentiated adenocarcinoma in 1 (3/15)). Based on the diagnostic criteria, 6 patients (6/15, 40 %) had ≥ 50 % myometrial invasion, and 9 patients (9/15, 60 %) had < 50 % myometrial invasion. According to the pathological grade, 53.33 % (8/15) of patients had pathologically confirmed poor differentiation, and 46.67 % (7/15) had good/moderate differentiation. Pelvic and/or paraaortic lymph node sampling was performed in 93.33 % (14/15) of patients. The mean time interval between MRI and hysterectomy was 7.33 days (range 1–30 days).

**Table 1:** Patient characteristics of 15 patients with cervical cancer at initial diagnosis

Characteristics	Value
**Total patients (n)**	15
**Mean age (years)**	50.20 ± 9.03 years (35 ~ 65 years)
**Menopausal status**	
** Premenopausal**	6
**Postmenopausal**	9
**Hispathologic subtype**	
**Endometrioid adenocarcinoma**	12
**Clear cell adenocarcinoma**	1
**Serous papillary adenocarcinoma**	1
**Low-undifferentiated adenocarcinoma**	1
**FIGO stage**	
**IA**	8
**IB**	2
**II**	2
**III**	3
**Pathological grade**	
**Well/moderately differentiation**	7
**Poorly differentiation**	8
**Myometrial invasion**	
**Shallow invasion**	9
**Deep invasion**	6

*FIGO *International Federation of Gynecology and Obstetrics

### Tumor stiffness predicts the aggressiveness of endometrial cancer

**Fig. 2:**
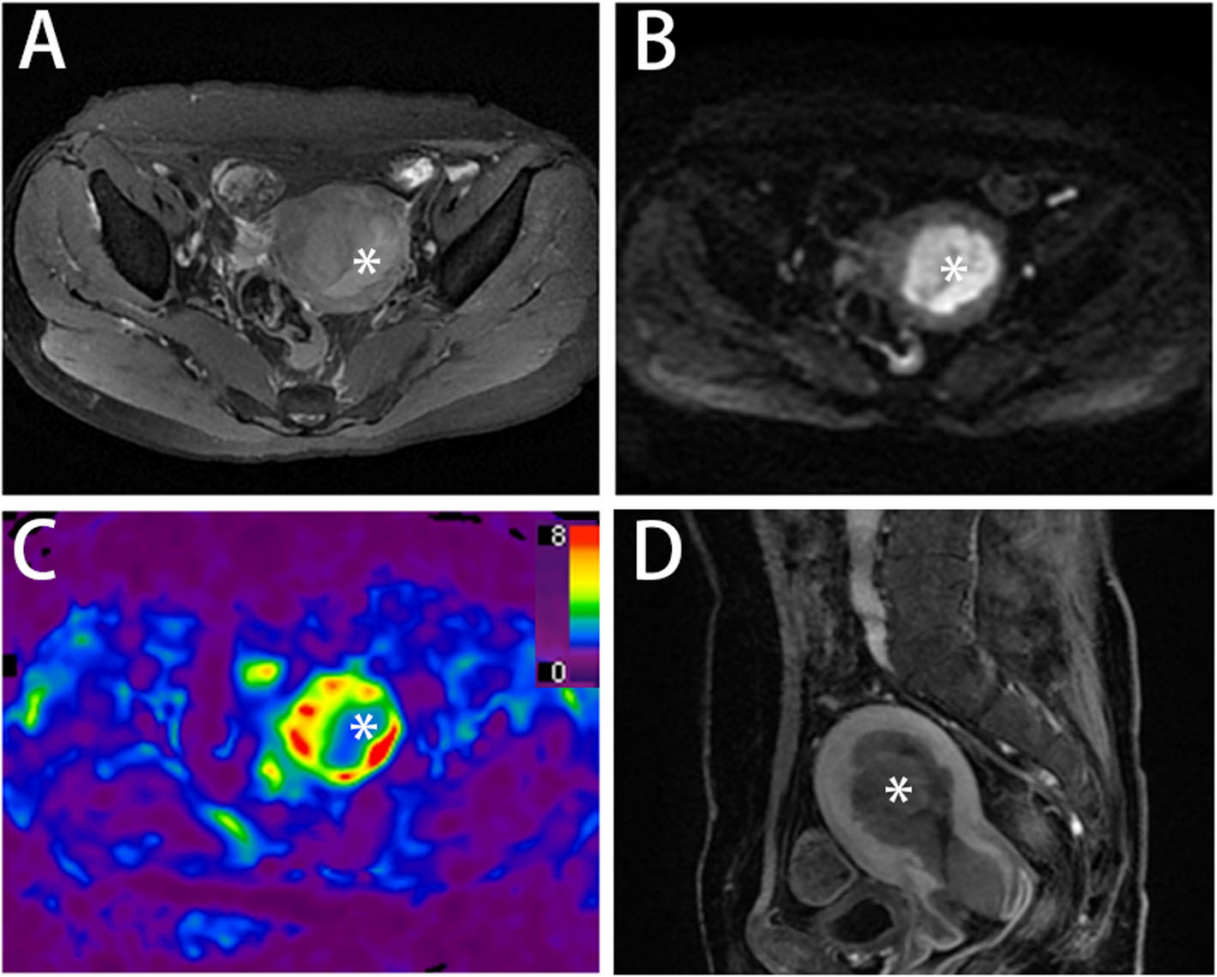
A 55-year-old woman presented with abnormal vaginal bleeding and underwent pelvic MRI and MRE scans. Axial T2-weighted image (**A**), diffusion-weighted image (**B**), and sagittal T1-weighted image with contrast (**D**) showed a mass (4.5×3.6 cm, asterisk) in the uterus. Tumor stiffness was 3.08 kPa measured by 3D MRE elastograms (**C**) at 60 Hz. Serous papillary adenocarcinoma (Type II) with deep myometrial invasion was surgically confirmed

**Fig. 3:**
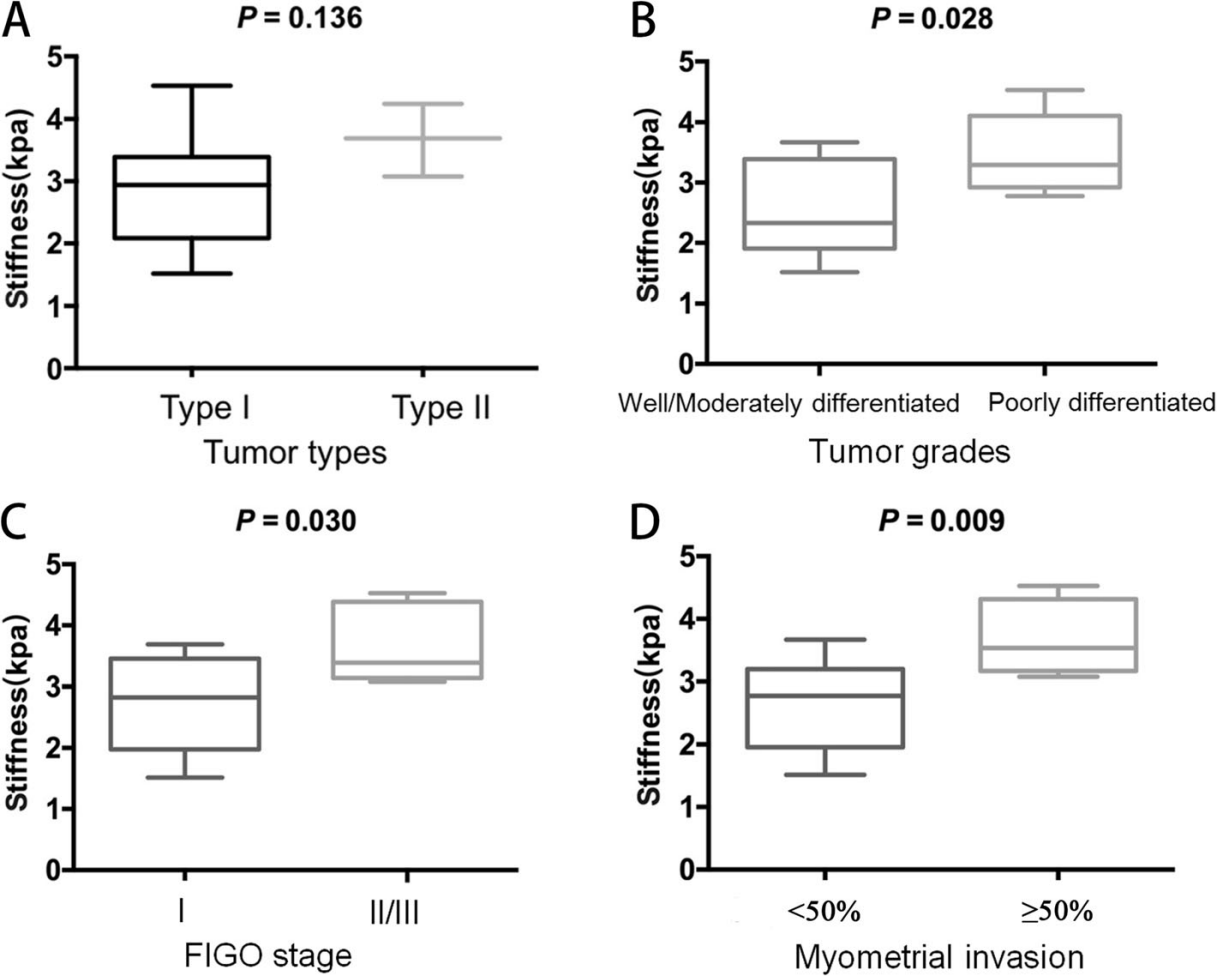
Comparison of the stiffness values between different EC groups

**Fig. 4:**
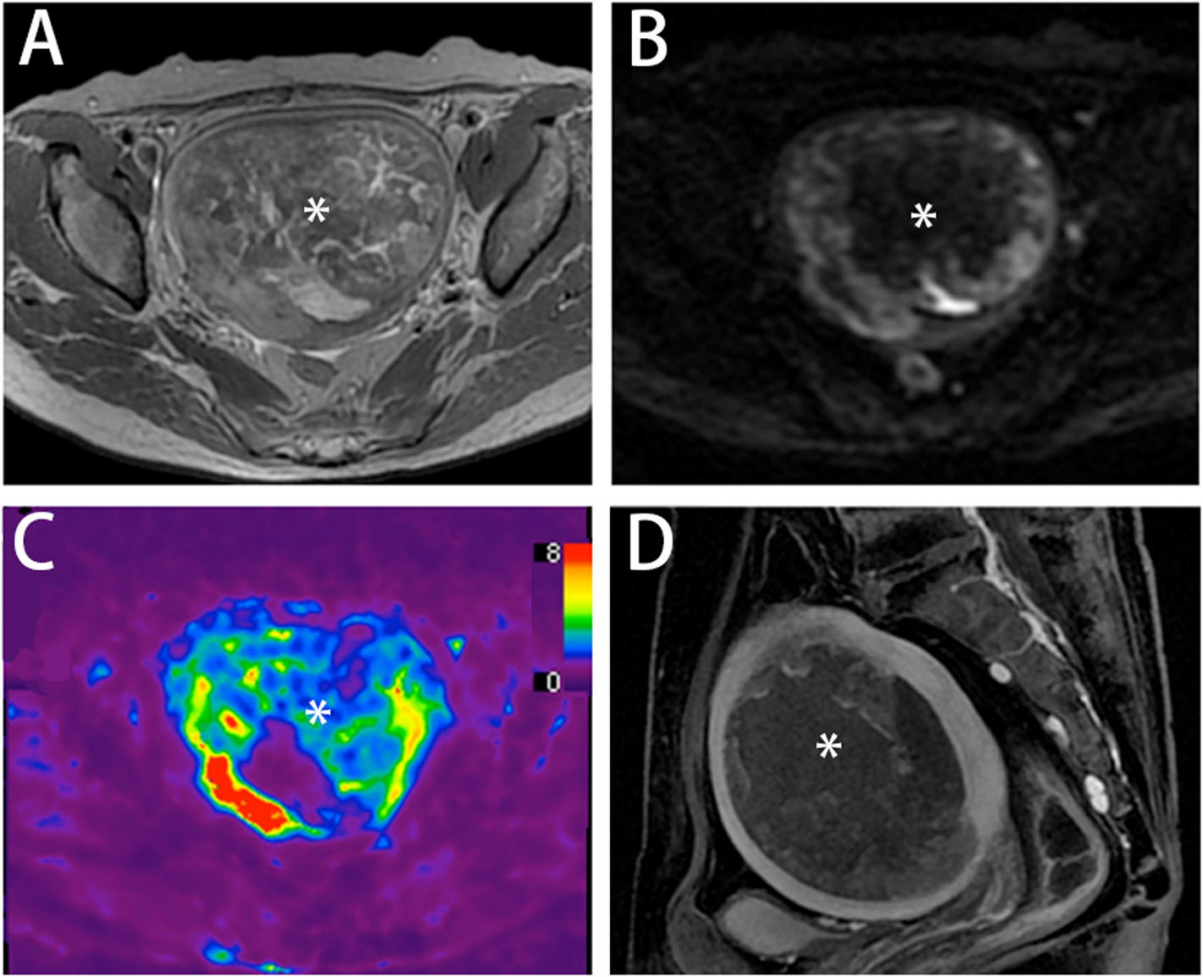
A 61-year-old woman presented with lower abdominal pain and underwent pelvic MRI and MRE scans. Axial T2-weighted image (**A**), diffusion-weighted image (**B**), and sagittal T1-weighted image with contrast (**D**) showed a mass (6.1×4.2 cm, asterisk) in the uterus. Tumor stiffness was 3.38 kPa measured by 3D MRE elastograms (**C**) at 60 Hz. Poorly differentiated endometrial adenocarcinoma (Type I) with deep myometrial invasion was surgically confirmed

**Fig. 5:**
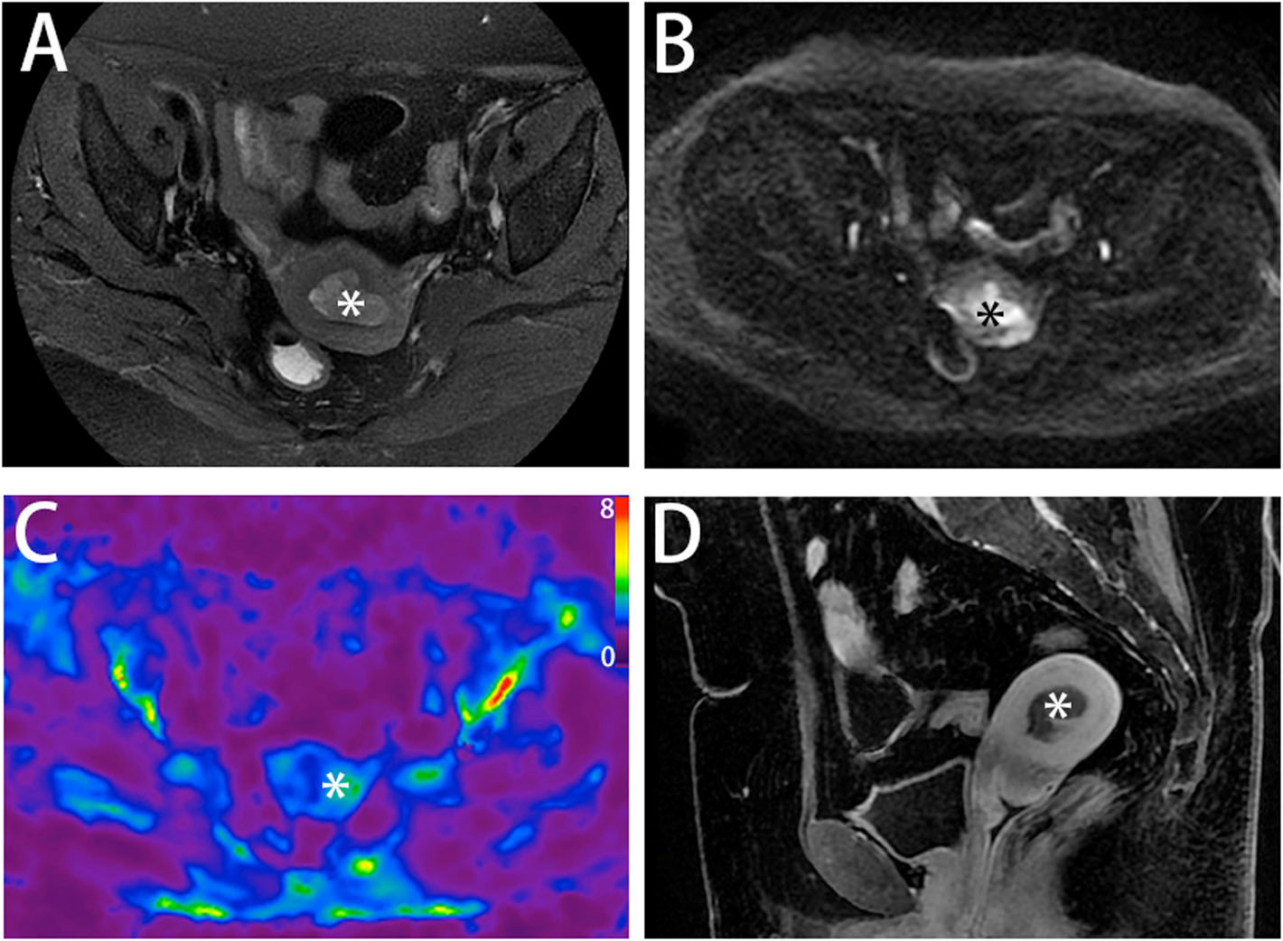
A 35-year-old woman presented with abnormal vaginal bleeding and underwent pelvic MRI and MRE scans. Axial T2-weighted image (**A**), diffusion-weighted image (**B**), and sagittal T1-weighted image with contrast (**D**) showed a mass (2.8×2.7 cm, asterisk) in the uterus. Tumor stiffness was 2.39 kPa measured by 3D MRE elastograms (**C**) at 60 Hz. Well-differentiated endometrial adenocarcinoma (Type I) with superficial myometrial invasion was surgically confirmed

The categorizations of endometrial cancer with corresponding stiffness values are detailed in Table 2. The tumor stiffness value in type II carcinoma (Fig. 2) was higher than that in type I carcinoma (3.67 ± 0.59 kPa vs. 2.88 ± 0.85 kPa, Fig. 3 A), but the difference was not significant (*p =* 0.136). The stiffness value of EC with ≥ 50 % myometrial invasion was significantly higher than that of EC with < 50 % myometrial invasion (3.68 ± 0.59 kPa vs. 2.61 ± 0.72 kPa, *p =* 0.009, Fig. 3 D). The cutoff value of stiffness to discriminate deep myometrial invasion of EC (Fig. 4) from superficial myometrial invasion of EC was determined to be 3.04 kPa with a sensitivity of 100 %, specificity of 77.8 %, and ROC curve area of 0.889 (95 % CI: 0.717-1.000). The stiffness value of poorly differentiated EC was significantly higher than that of well/moderately differentiated EC (3.47 ± 0.64 kPa vs. 2.55 ± 0.*82* kPa, *p* = 0.028, Fig. 3 B). The cutoff value of stiffness to discriminate poorly differentiated EC from well/moderately differentiated EC (Fig. 5) was determined to be 3.04 kPa, with a sensitivity of 75 %, specificity of 71.4 %, and ROC curve area of 0.786 (95 % CI: 0.541-1.000). The stiffness value of FIGO stage II/III EC was significantly higher than that of FIGO stage I EC (3.69 ± 0.65 kPa vs. 2.72 ± 0.76 kPa, *p* = 0.030, Fig. 3 C). The cutoff value of stiffness to discriminate FIGO stage I EC from FIGO stage II/III EC was determined at 3.04 kPa, with a sensitivity of 100 %, specificity of 70 %, and ROC curve area of 0.840 (95 % CI: 0.635-1.000). The tumor size of FIGO stage II/III EC was significantly higher than that of FIGO stage I EC (8.24 ± 4.12 cm vs. 4.09 ± 2.53 cm, *p* = 0.030), but there were no significant differences in tumor size between the other subgroups (tumor grade, histological subtype, and myometrial invasiveness). For DWI, the ADC value showed no significant differences between any subgroups.

**Table 2:** Categorization of endometrial cancer with corresponding stiffness, tumor size, and ADC values

Category	n	MRE		Tumor size		DWI	
		**Stiffness (kPa)**	***p*value**	**Diameter(cm)**	***p*value**	**ADC(×10**^**− 3**^**mm**^**2**^**/s)**	***p*value**
**Tumor types**			0.136		0.973		0.777
** Type I**	12	2.88 ± 0.85		5.45 ± 3.92		753.15 ± 84.10	
**Type II**	3	3.67 ± 0.58		5.54 ± 2.63		737.66 ± 77.52	
**Tumor grades**			0.028*		0.109		0.726
**Well/Moderately differentiated**	7	2.55 ± 0.82		3.86 ± 2.07		758.24 ± 72.51	
**Poorly differentiated**	8	3.47 ± 0.64		6.88 ± 4.20		742.88 ± 91.01	
**FIGO stage**			0.030*		0.030*		0.716
**I**	10	2.72 ± 0.76		4.09 ± 2.53		755.68 ± 85.85	
**II/III**	5	3.69 ± 0.65		8.24 ± 4.12		738.78 ± 76.02	
**Myometrial invasion**			0.009*		0.089		0.979
<**50 %**	9	2.61 ± 0.72		3.91 ± 1.85		750.51 ± 93.52	
**≥ 50 %**	6	3.68 ± 0.59		7.81 ± 4.50		749.35 ± 63.91	

*MRE *magnetic resonance elastography, *DWI* diffusion-weighted imaging, *ADC* Apparent diffusion coefficient, *FIGO* International Federation of Gynecology and Obstetrics, * p < 0.05

In addition, the interobserver variability of tumor stiffness was low, with intraclass correlation coefficients of 0.89 to 0.94 and a minimum detectable change of 0.01 to 0.2 kPa for the different tumor stiffness measurements.

## Discussion

To our knowledge, this is the first study to use 3D MRE to assess the aggressiveness of EC. In our preliminary study, we demonstrated that preoperative tumor stiffness as measured by 3D MRE could predict tumor aggressiveness, including grade of differentiation, FIGO stage, and depth of myometrial invasion of EC, suggesting that this parameter may serve as a noninvasive imaging biomarker for preoperative risk stratification to guide surgical treatment and adjuvant therapy.

In our series, we found that the stiffness value of EC with deep myometrial invasion was significantly higher than that of EC with superficial myometrial invasion, with a cutoff value of 3.04 kPa to distinguish EC with deep myometrial invasion from EC with superficial myometrial invasion. However, the role of stiffness in modulating the growth and progression of EC remains poorly defined. Previous studies have demonstrated that several factors may contribute to increased malignant stiffness, including collagen deposition in the extracellular matrix, high cellularity, abnormal perfusion, and increased interstitial fluid pressure from the altered vasculature [22, 23]. Currently, increased matrix stiffness has been shown to promote cellular proliferation in glioma, hepatocellular carcinoma and breast cancer cells, which is closely related to tumor vascular invasion [24–26]. Furthermore, previous studies have also shown that an increased tumor interstitial fluid pressure, which is possibly associated with leaky tumor vasculature, lack of lymphatic vessels, and abundance of angiogenesis-stimulating cytokines, may increase cancer cell invasion and then promote tumor progression [22, 23]. These changes can be quantified with elastography noninvasively, providing useful information about the tumor microenvironment, particularly the ECM, and its modification by stromal cells [27].

Early-stage EC shows a good prognosis, and the 5-y survival rate is reported to be more than 90 % in patients with stage Ia or Ib disease [3]. However, patients with advanced disease have significantly worse survival. In our study, the stiffness value of FIGO stage II/III EC was significantly higher than that of FIGO stage I EC. In addition, the stiffness values of poorly differentiated EC were significantly higher than those for well/moderately differentiated tumors. Our results regarding tumor grade were consistent with previous studies on hepatocellular carcinoma, which showed that the stiffness in poorly differentiated hepatocellular carcinoma was higher than that in well/moderately differentiated hepatocellular carcinoma [18]. Two possible explanations for this result are discussed. According to the FIGO grading system, when EC cells transform from a glandular pattern into solid sheets of cells, the tumor is considered to be poorly differentiated [28]. However, this may be attributed to the higher cell density of poorly differentiated ECs [28, 29]. It is well known that the degree of EC differentiation is closely related to the cell growth rate. Poorly differentiated ECs grow faster, which leads to an increase in cell density [14]. A previous study of prostate cancer showed that stiffness was significantly related to the Gleason score, indicating that stiffness can be used to predict tumor grade [29]. MRE studies of hepatocellular carcinoma and other malignancies also revealed that stiffness measured by MRE can be used as a quantitative biomarker for the noninvasive prediction of pathological characteristics and outcomes [11–18]. Recent studies also showed that evaluating liver tumor stiffness can help differentiate benign and malignant liver focal lesions, predict tumor recurrence after hepatic resection, and predict the response to sorafenib-treated advanced hepatocellular carcinoma, which is in accordance with our study [11, 12, 30].

Two distinct types of EC based on histological and molecular characteristics have been described in a previous study, and type II EC is usually less differentiated than type I EC and is more prone to infiltration and metastasis [31, 32]. Our study showed a trend toward a higher tumor stiffness value in type II than in type I, which was consistent with the pathological characteristics and biological behavior of the two types. However, the differences in stiffness values between the two types of EC were not statistically significant since the sample size of type II EC in our study was relatively small (only 3), which might hinder the ability to obtain statistically significant results. Further studies with larger populations are warranted to confirm the value of tumor stiffness in the evaluation of EC subtype. In our study, we also demonstrated that the tumor size of FIGO stage II/III EC was significantly higher than that of FIGO stage I EC, which was in accordance with other studies [7]. For DWI, the ADC value showed no significant differences between the subgroups. Our results were not consistent with those of previous studies [33, 34]. The discrepancy between these results may be attributed to the small sample size of our study. Further prospective cohort studies with large sample sizes should be performed to explore the relationship between DWI and MRE and determine which of these modalities are more important in preoperatively evaluating the aggressiveness of EC. For any quantitative parameter, high accuracy and reproducibility are essential. Due to its cost-effectiveness and lack of radiation, ultrasound-based elastography is the most widely used and validated imaging method to measure tumor stiffness in women with EC [35, 36]. However, ultrasound-based elastography has several limitations, such as operator dependence, interobserver variation, and measurement difficulties in patients with severe obesity or ascites. In our study, we found that tumor stiffness was measured with very low interobserver variability, regardless of the previous experience of readers, which was an obvious advantage over ultrasound elastography. Thus, tumor stiffness seems to represent a robust biomarker that is promising for predicting tumor aggressiveness in EC.

Despite our promising results, our preliminary study does have several limitations. First, this was a retrospective study performed at a single institution, and the sample size was relatively small. Thus, our findings need to be reproduced in larger patient series with longer follow-up and evaluation of prognostic information to confirm the possible value of MRE as a preoperative biomarker in EC. Second, as a confounding factor of bias, menopausal status may influence our results. In our small cohort study, 40 % of the patients were premenopausal. A previous study showed that stiffness changed in the uterine corpus during the menstrual cycle, which was related to the anatomic and functional alteration of both the myometrium and the endometrium [19]. Future studies should include information regarding menstruation and menopause status to investigate the correlation between female hormonal state and tumor stiffness of EC. Third, given the heterogeneity of EC [37], the tumor stiffness used in our study may not adequately reflect tumor heterogeneity, and some form of histogram analysis covering the whole tumor might be useful in future studies. Fourth, we did not compare the diagnostic performance of MRE with that of conventional MRI. Although we believe that the combination of multiparametric MR offers more diagnostic value than simple MR alone, it would still be meaningful to study this topic. Fifth, we excluded patients with lesions smaller than 2 cm in diameter with a stiffness that may not be measured accurately, as using a 60-Hz vibration frequency may be less effective in resolving small stiff tumors due to the long shear wavelength. In future work, the use of higher vibration frequencies may have to be considered for smaller tumors.

## Conclusions

In conclusion, preoperative tumor stiffness as measured by 3D MRE could predict tumor aggressiveness and may be a strong prognostic factor in EC. Preoperative 3D MRE as part of a multiparametric MR protocol may, thus, serve as a noninvasive imaging biomarker for diagnosis and appropriate therapeutic strategy determination for patients with EC.

## Data Availability

The dataset supporting the conclusions of this article is included within the article. Data and materials associated with the current study are available from the corresponding author upon reasonable request.

## Abbreviations

EC: Endometrial carcinoma
FIGO: International Federation of Gynecology and Obstetrics
3D: Three-dimensional
MRE: Magnetic resonance elastography
ROC: Receiver operating characteristic
MRI: Magnetic resonance imaging
ROI: Region of interest
kPa: Kilopascals
DWI: Diffusion-weighted imaging
ADC: Apparent diffusion coefficient
